# Iatrogenic Chest Wall Arteriovenous Malformation Following Chest Tube Placement: A Case Report

**DOI:** 10.7759/cureus.98925

**Published:** 2025-12-10

**Authors:** Gerilyn Boyle, Gaston Becherano, Robert H Mbilinyi, Gary Schwartz

**Affiliations:** 1 Medical School, Texas A&amp;M University Naresh K. Vashisht College of Medicine, Bryan, USA; 2 Cardiothoracic Surgery, Baylor University Medical Center, Dallas, USA

**Keywords:** arteriovenous malformation embolization, arteriovenous malformations, chest wall avm, chest wall surgery, thoracoscopy, traumatic avm

## Abstract

Arteriovenous malformations (AVMs) are vascular anomalies characterized by direct arterial-to-venous connections that bypass the capillary system. Chest‑wall AVMs are rare, with only few reports of iatrogenic AVMs following chest‑tube placement. We describe a large, symptomatic chest‑wall AVM developing at the site of prior chest‑tube insertion, highlighting diagnostic, operative, and follow‑up strategies.

We present a case of a 37-year-old male patient with a large, symptomatic chest wall AVM centered around a scar from a prior chest tube placement. A thorough preoperative assessment was conducted, including imaging and a biopsy, which confirmed the diagnosis. Given the lesion’s hypervascularity, the risk of significant bleeding during biopsy was carefully considered, and the procedure was performed with interventional radiology support to minimize complications. The decision was made to proceed with targeted embolization of the lesion, significantly reducing intraoperative hemorrhage risk and facilitating safe subsequent surgical intervention. A combined open and thoracoscopic surgical approach led to successful resection of the lesion.

This report highlights that although the majority of AVMs are congenital, there is a significant potential for iatrogenic AVM formation, particularly following invasive procedures such as chest tube placement. This case underscores the importance of a multidisciplinary approach in diagnosing and managing complex vascular anomalies and highlights lessons learned in careful chest tube placement to avoid vascular injury.

## Introduction

Arteriovenous malformations (AVMs) are high-flow vascular anomalies characterized by abnormal connections between arteries and veins that bypass the capillary network. These malformations can lead to complications such as hemorrhage, tissue ischemia, mass effect on surrounding structures, and high-output cardiac failure due to increased blood flow [1,2]. AVMs are most commonly found in the head, neck, and extremities but are rarely located in the chest wall, making such cases particularly unique and diagnostically challenging [1,3,4]. The unusual location, size, and complexity of chest wall AVMs add to the difficulty in their diagnosis and management. Understanding their unique challenges is key to ensure effective treatment planning and favorable patient outcomes [1,4]. The vast majority of AVMs are congenital, arising from developmental errors in angiogenesis. However, acquired forms of AVMs, often associated with trauma or surgery, have also been recognized [2,5]. Acquired AVMs can emerge years after the initial injury due to chronic vascular remodeling, implicating both mechanical and physiological factors in their pathogenesis [6,7]. We present a unique case of an iatrogenic AVM in a 37-year-old male patient, likely associated with prior chest tube placement for a primary spontaneous pneumothorax. This case highlights the complexity of managing AVMs in the chest wall due to their size, vascularity, and proximity to critical structures. A multidisciplinary management approach involving imaging modalities such as computed tomography angiography, embolization, cryoablation, and surgical resection is essential for achieving favorable outcomes in such rare and challenging cases.

This article was previously presented as a poster at the 11th Annual BUMC Medical Education Research Forum on April 8, 2025.

## Case presentation

**Figure 1:**
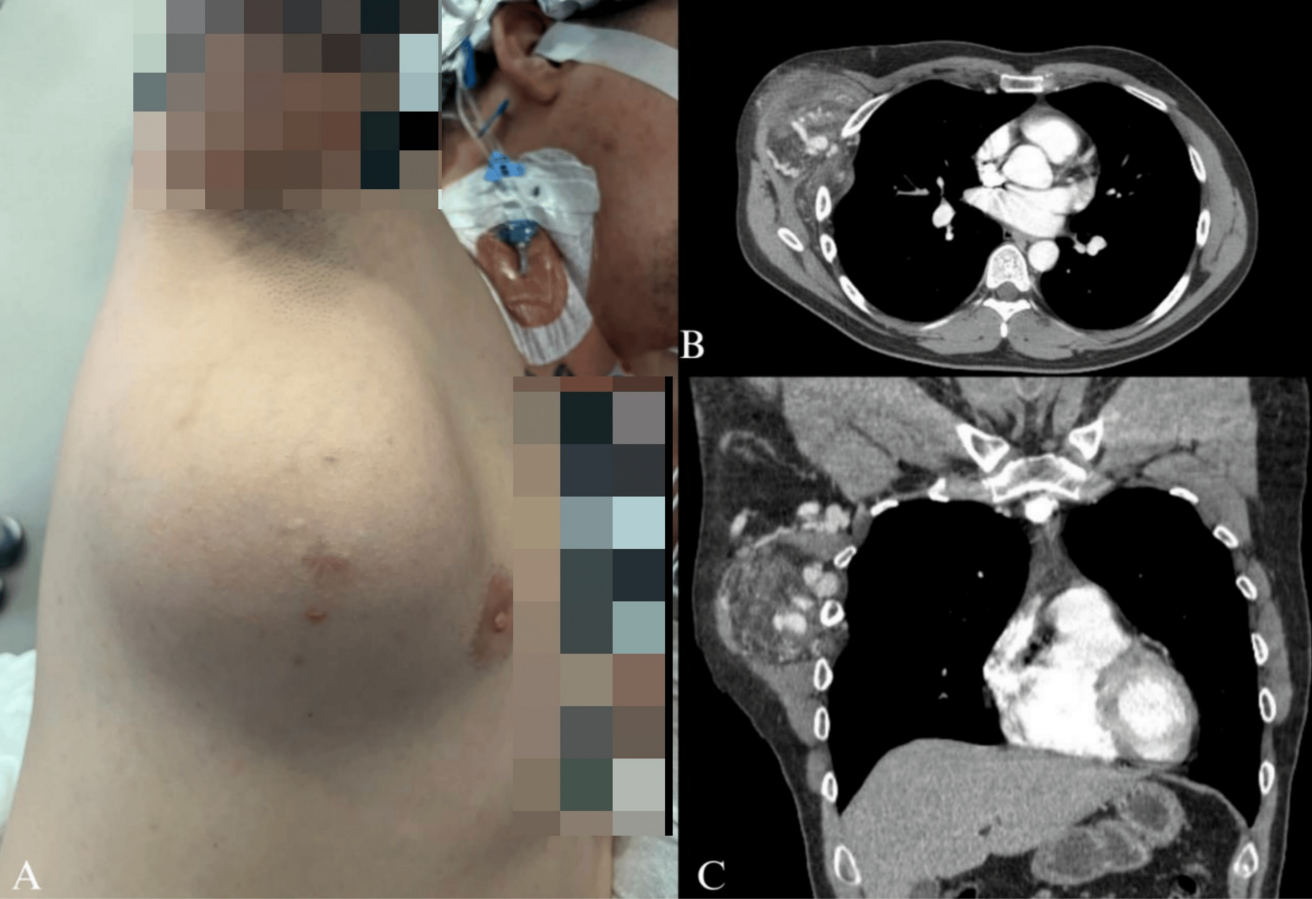
Clinical and Radiographic Presentation of the Chest Wall AVM. (A) Clinical image showing a prominent chest wall mass in the right anterior thoracic region, centered around a scar from a prior chest tube placement. (B) Axial CT scan with contrast demonstrating a heterogeneous, hypervascular lesion in the right lateral chest wall. The lesion involves the serratus anterior muscle and shows arterial and venous components, with adjacent tissue displacement. (C) Coronal CT view further illustrating the vascular malformation with prominent feeding arteries, including the right subclavian artery and internal mammary artery, and draining veins. AVM: Arteriovenous Malformation

A 37-year-old man presented with a progressively enlarging right chest wall mass causing discomfort. Physical examination revealed a well-appearing male with a palpable, non-tender mass in the right axillary region (Figure 1A). The mass, centered around a scar from a prior 2 chest tube placement for a primary spontaneous pneumothorax, had gradually increased in size over several years. Computed tomography (CT) of the chest with contrast demonstrated a highly vascular 13 × 8 cm mass involving the serratus anterior muscle, displacing adjacent tissues and extending between intercostal spaces (Figures 1B-1C). The right subclavian artery was enlarged, feeding a 3.7 cm cluster of vessels in the axilla. The right internal mammary artery and vein were enlarged compared to the left, and direct invasion through the chest wall into the extrapleural space was noted between the anterolateral right third and fourth intercostal spaces. Subtle periosteal thickening of the ventral margin of the right fourth rib suggested chronic involvement. These findings were consistent with an AVM.

A biopsy of the mass was done to rule out malignancy. Given the lesion’s hypervascularity, the risk of significant bleeding during biopsy was carefully considered, and the procedure was performed with interventional radiology (IR) support to minimize complications. Multidisciplinary consultation with vascular surgery, IR, and thoracic surgery confirmed the AVM diagnosis. A staged treatment plan was devised to mitigate the risks associated with this large and complex lesion.

**Figure 2:**
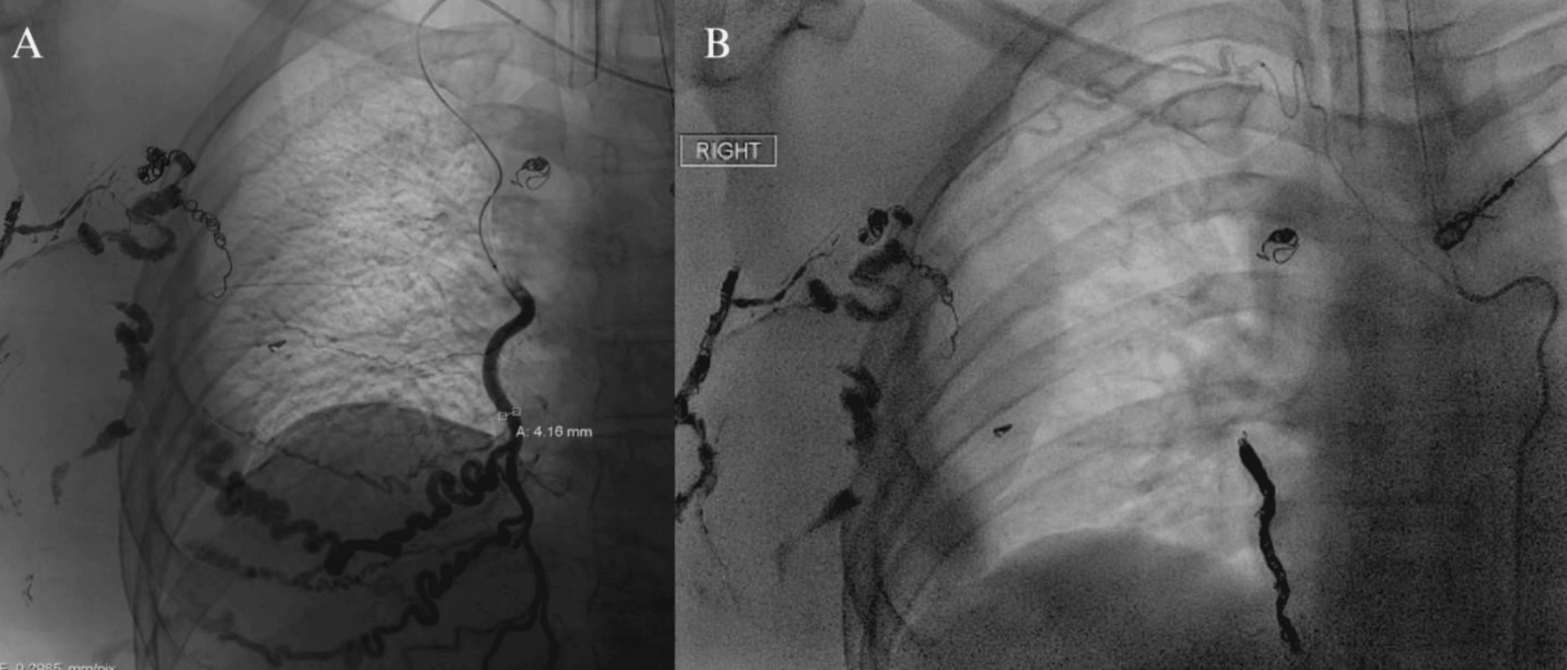
Preoperative Embolization of the Chest Wall AVM. (A) Digital subtraction angiography showing the AVM’s arterial supply from branches of the right internal mammary artery and anterior intercostal arteries. (B) Post-embolization angiography demonstrating successful coil embolization with significant stasis of arterial inflow. AVM: Arteriovenous Malformation

To reduce intraoperative hemorrhage risk, preoperative percutaneous embolization targeted large feeding vessels, including branches of the right internal mammary artery and anterior intercostal arteries. Coil embolization was successfully performed, achieving significant stasis of arterial inflow as confirmed by digital subtraction angiography (Figures 2A-2B). One week after embolization, the patient underwent open surgical excision of the mass. Thoracoscopy was utilized during the procedure to visualize the internal aspect of the mass, where it interdigitated between the ribs, aiding in the open resection while sparing rib resection. The lesion was resected en bloc, including its intrapleural component. Reconstruction involved the advancement of serratus myocutaneous flaps, and intercostal 3 nerve cryoablation was performed for postoperative analgesia.

**Figure 3:**
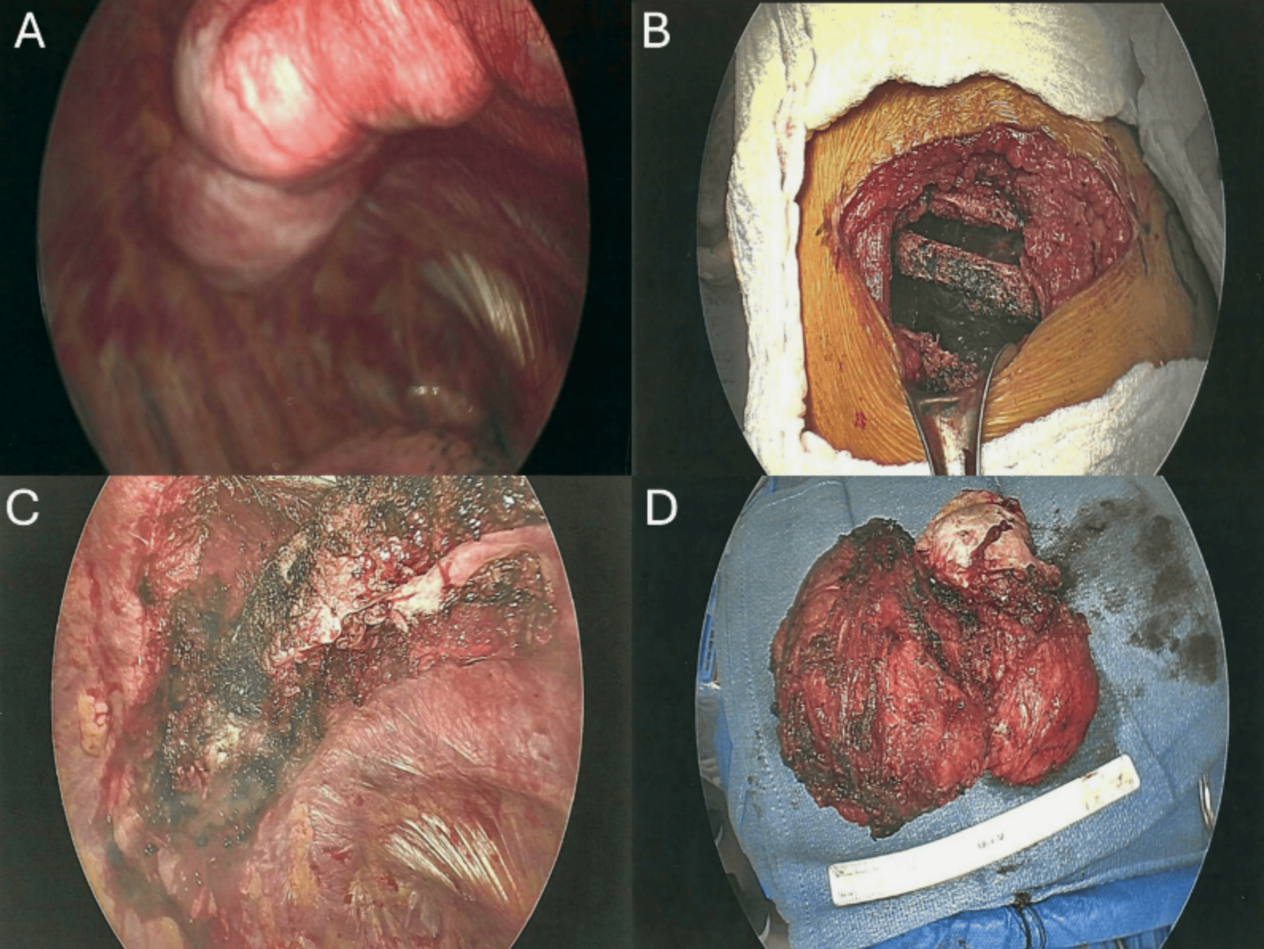
Intraoperative and Gross Specimen Findings of the Chest Wall AVM. (A) Thoracoscopic views showing the AVM interdigitating between ribs and its extensive vascularity. (B) External surgical view after resection, showing the resected serratus anterior and intercostal muscles with spared ribs. (C) Post-resection thorascopic view after en bloc removal, with reconstruction using serratus muscle flaps and intercostal nerve cryoablation for pain control. (D) Gross specimen of the 16 × 12 × 7.5 cm, 631 g AVM. AVM: Arteriovenous Malformation

The resected specimen weighed 631 grams and measured 16.0 × 12.5 × 7.5 cm (Figure 3). It consisted of fibroadipose tissue and skeletal muscle with a slightly whorled to trabecular appearance. Numerous malformed, thick-walled vessels were observed, with lumina measuring up to 1.4 cm and organized thrombi. Findings were consistent with an AVM with no evidence of malignancy.

The patient tolerated the procedure well and had an uneventful recovery. Four days postoperatively, he was discharged home in good condition, with marked improvement in symptoms. Postoperative surveillance with contrast-enhanced CT was performed at three, six, and 12 months, demonstrating no recurrence or revascularization. The patient reported significant improvement in functional capacity and pain.

## Discussion

Chest wall AVMs are rare vascular anomalies that can be difficult to diagnose and treat, particularly when they become large and chronic. This case, involving a 16 × 12 × 7.5 cm lesion, highlights the rarity of an extensive chest wall AVM. Comparable reports, such as a 5.0 × 14.0 × 10.8 cm lesion in a 55-year-old male patient, show how these anomalies can remain undetected for prolonged periods [1] and potentially lead to extensive collateral circulation, anatomic distortion, and clinical presentations that mimic other pathologies [7,8].

Although many AVMs are congenital, increasing evidence indicates that trauma or surgical interventions can contribute to acquired or iatrogenic lesions [7-9]. In this case, the close association between the lesion and the prior chest tube scar suggests that vascular injury during thoracostomy led to abnormal arterial-venous connections that gradually expanded through chronic remodeling. The mechanism likely involves simultaneous damage to adjacent intercostal arteries and veins, creating the substrate for progressive arteriovenous shunting [3,7,9]. This highlights a critical lesson for clinicians: careful chest tube placement is essential to minimize vascular injury, especially in the anterolateral thoracic wall, where intercostal bundles are most vulnerable. Using blunt dissection to access the pleural space, avoiding excessive force [4], and selecting the appropriate intercostal space can help reduce the risk. Furthermore, any patient who develops persistent or unexplained chest wall swelling, pulsatility, or bruit following chest tube placement should undergo early imaging to detect potential vascular complications before they progress [3,9].

High-quality imaging and histopathologic assessment are crucial for differentiating AVMs from neoplasms or other vascular entities. Contrast-enhanced CT can reveal serpiginous vessels, enlarged feeding arteries, and periosteal thickening, suggesting a long-standing, high-flow process [1,10]. Even when imaging strongly points to an AVM, biopsy may be necessary to exclude malignancy. In this patient, benign fibroconnective tissue and skeletal muscle with increased vascularity confirmed the diagnosis. Histology often demonstrates focal loss of the internal elastic lamina in arteries and thickened venous walls [3,11], highlighting the importance of tissue confirmation when imaging features could otherwise be misinterpreted as malignant [12,13].

Managing large or atypical AVMs, especially those with complex vascular architecture or iatrogenic origins, benefits significantly from a multidisciplinary approach. Collaboration among thoracic surgery, vascular surgery, and IR enabled preoperative embolization followed by surgical resection, minimizing intraoperative bleeding and improving outcomes [1,7,8]. Notably, thoracoscopy served as an adjunct to open resection, aiding visualization of intrathoracic components without requiring a wholly minimally invasive approach. The successful use of serratus muscle advancement flaps and intercostal nerve cryoablation further demonstrates how reconstruction and pain management can be tailored to individual patient needs.

Long-term follow-up is critical, given the potential for recurrence or revascularization. Early detection of recurrent or newly forming feeding vessels is vital, as is ongoing evaluation of functional status. In this case, the absence of recurrence and marked improvement in pain and activity highlight the effectiveness of a patient-centered, stepwise intervention. Routine imaging surveillance and clinical assessments offer additional insight into the durability of these treatments [5]. However, as with all single-patient case reports, these observations cannot be generalized broadly and should be interpreted within the context of an individual clinical experience. Despite advances in imaging, embolization, and surgical techniques, no standardized guidelines exist for managing large, chronic, and potentially iatrogenic chest wall AVMs. Thoracoscopic or hybrid approaches, while promising, require more extensive evaluation to clarify their applicability and impact on morbidity. Research into genetic, molecular, and hemodynamic factors underlying trauma-induced AVM pathogenesis may reveal novel therapeutic targets and refine risk stratification. Larger cohorts with extended follow-up are also needed to deepen understanding of recurrence patterns and guide long-term strategies.

## Conclusions

This case highlights the importance of recognizing that AVMs can arise from non-congenital, iatrogenic causes such as prior chest tube placement and that these lesions can become large, symptomatic, and complex to manage over time. Successful treatment relied on a multidisciplinary, staged approach that integrated careful diagnostic evaluation, preoperative embolization, thoracoscopic assistance for surgical resection, and structured postoperative surveillance. Beyond technical management, this case underscores the need for vigilance in chest tube placement to avoid vascular injury, for early consideration of AVMs when patients present with progressive chest wall masses, and for individualized reconstruction and pain-control strategies to optimize recovery. Ultimately, this experience demonstrates how thoughtful planning and collaboration across specialties can improve outcomes in rare but formidable vascular malformations.
